# A Nonrandomized Clinical Trial Investigating Keratinocyte Growth Factor‐Hair Serum for the Prevention of Chemotherapy‐Induced Alopecia

**DOI:** 10.1111/jocd.70797

**Published:** 2026-03-16

**Authors:** Katherine Mann, Preethika Potluri, Emma E. Paul, Jennifer M. Segar, Sima Ehsani, Denise Roe, Pavani Chalasani

**Affiliations:** ^1^ George Washington School of Medicine and Health Sciences Washington DC USA; ^2^ University of Arizona Cancer Center Tucson Arizona USA; ^3^ Banner University Medical Center Tucson Arizona USA

**Keywords:** breast cancer, chemotherapy‐induced alopecia, keratinocyte growth factor‐hair serum

## Abstract

**Introduction:**

Chemotherapy‐induced alopecia (CIA) is known to have a significant psychological and quality of life impact. Although cold caps have been shown to prevent CIA, expense and extension of treatment durations are barriers for routine clinical use. Keratinocyte growth factor (KGF) has been shown to have cytoprotective effects on human hair follicles and reduce alopecia in preclinical models. We hypothesized that KGF‐hair serum (KGF‐HS) will prevent CIA.

**Methods:**

We conducted a Simon two‐stage, single‐arm clinical study in women with early‐stage breast cancer (ESBC) scheduled to receive at least four cycles of chemotherapy. The primary outcome was preservation of hair after chemotherapy, whereas secondary measures included patient‐reported wig use, comfort, and validated quality‐of‐life instruments (EORTC QLQ‐C30, HADS, and BIS).

**Results:**

Twenty patients were evaluable for the primary end point. None achieved meaningful hair preservation. The average duration of treatment of KGF‐HS application was 4.6 weeks.

**Conclusion:**

In this study of women with ESBC receiving chemotherapy, using the KGF‐HS did not prevent CIA. There was no statistical difference pre‐ and post‐study BIS, HADS, and EORTC‐30 scores. Application of the KGF‐HS was reported to be easy, with minimal discomfort, and a non‐oily appearance. Patients' ease of use and acceptability of a topical agent for CIA further supports the development of new agents for a more practical and affordable alternative to scalp cooling.

**Trial Registration:**

clinicaltrials.gov: NCT04554732

## 1 Introduction

Chemotherapy‐induced alopecia (CIA) is a common adverse event following cytotoxic treatments. It occurs in 65% of cancer patients and has significant negative mental health and quality of life impacts [1]. 75% of cancer patients report it as the most feared complication and as many as 10% of patients reject chemotherapy due to CIA [2]. CIA is typically reversible with hair regrowth seen 3–6 months after the chemotherapy treatment ends. However, the regrown hair may be of different quality, color, and thickness, and can continue to have a significant psychological burden on cancer patients as 73% of women say they never regained their self‐confidence even after the regrowth of their hair [3].

Current treatment options for CIA are limited. In recent years, scalp cooling has been shown to reduce CIA. Scalp cooling involves placing a tight‐fitting cap onto the patient's head and cooling the entire scalp before, during, and after chemotherapy. The mechanism of scalp cooling is based on cutaneous vasoconstriction and reducing blood flow to the hair follicles via cooling. This decreases chemotherapy delivery, thereby preventing hair loss [4]. Although clinical trials have shown the effectiveness of scalp cooling, in practice there are time and financial barriers. Scalp cooling requires the patient to wear the cap for 60 min prior to the chemotherapy infusion and again for 90–120 min after chemotherapy [5]. In addition, scalp cooling is also expensive as it is currently not covered by insurance. The average price of scalp cooling for patients ranges anywhere from $1000 to $8000. Due to these barriers, although scalp cooling has shown to be effective, it is not a practical solution for wide clinical application.

Keratinocyte growth factor (KGF) is a member of the fibroblast growth factor family and has been shown to be important in normal hair follicular growth, development, and differentiation [6]. KGF has a cytoprotective effect on hair cells and can increase regeneration on epithelial cells that had a toxic exposure [7]. Currently, KGF is used to prevent oral mucositis in chemotherapy patients as it increases epithelial cell resistance to toxic insult [8]. KGF has been shown to preserve hair follicles in as much as 50% of mice models when beginning 1 day prior to the onset of chemotherapy [6]. These data suggest that KGF might be effective in preventing chemotherapy‐induced hair loss. We studied a commercially available KGF‐Hair Serum (KGF‐HS) to prevent CIA. The purpose of our clinical trial was to assess the efficacy of KGF‐HS as a prophylactic treatment for CIA. It was hypothesized that in patients with CIA, the topical application of KGF‐HS will significantly reduce hair loss.

## 2 Materials and Methods

We designed a Simon two‐stage prospective study to assess KGF‐HS to prevent CIA. The study was approved by our institutional review board and registered on clinicaltrials.gov. All included patients gave written informed consent for clinical trial participation in compliance with the informed consent regulations in 21 CFR 50. The trial was also conducted in accordance with the Declaration of Helsinki for biomedical research involving human subjects and local regulatory requirements.

Key eligibility criteria were having early‐stage breast cancer (I–III) and scheduled but not begun at least four cycles of taxane and/or anthracycline‐based chemotherapy. Key exclusion criteria included any patients who were previously diagnosed with a hair loss disorder, scalp inflammatory condition, or KGF allergy.

KGF‐HS used for this study was secured from Skin Actives. Ingredients are water, Lactobacillus/Kelp ferment filtrate, PEG‐40 hydrogenated castor oil, propanediol, glycerin, *Serenoa serrulata* (saw palmetto) fruit extract, carnitine, niacinamide, *Cocos nucifera* (coconut) fruit juice, *Urtica dioica* (nettle) extract, *Boswellia serrata* extract, *Vitis vinifera* (grape) seed extract, ubiquinone, fucoidan, alanine, arginine, asparagine, aspartic acid, cysteine, glutamic acid, glutamine, glycine, histidine, isoleucine, leucine, lysine, methionine, phenylalanine, proline, serine, threonine, tryptophan, tyrosine, valine, biotin, choline chloride, cyanocobalamin, folic acid, inositol, calcium pantothenate, pyridoxine HCl, riboflavin, thiamine HCl, *Punica granatum* (pomegranate) seed oil, tocotrienols, tocopherols, astaxanthin, lycopene, xanthophyll, R‐alpha lipoic acid, beta‐carotene, sh‐polypeptide‐3, sh‐polypeptide‐107, superoxide dismutase, catalase, ammonium vanadate, ammonium molybdate, calcium chloride, copper sulfate, ferric nitrate, magnesium chloride, manganese sulfate, potassium chloride, sodium acetate, sodium chloride, sodium metasilicate, sodium phosphate, sodium pyruvate, disodium selenite, stannous chloride, zinc sulfate, adenine, thymidine, glucose, hydroxyethylpiperazine ethane sulfonic acid, thioctic acid, spermidine, xanthan gum, *Cyamopsis tetragonoloba* (guar) gum, calcium disodium EDTA, phenoxyethanol, caprylyl glycol, and sorbic acid.

**FIGURE 1:**
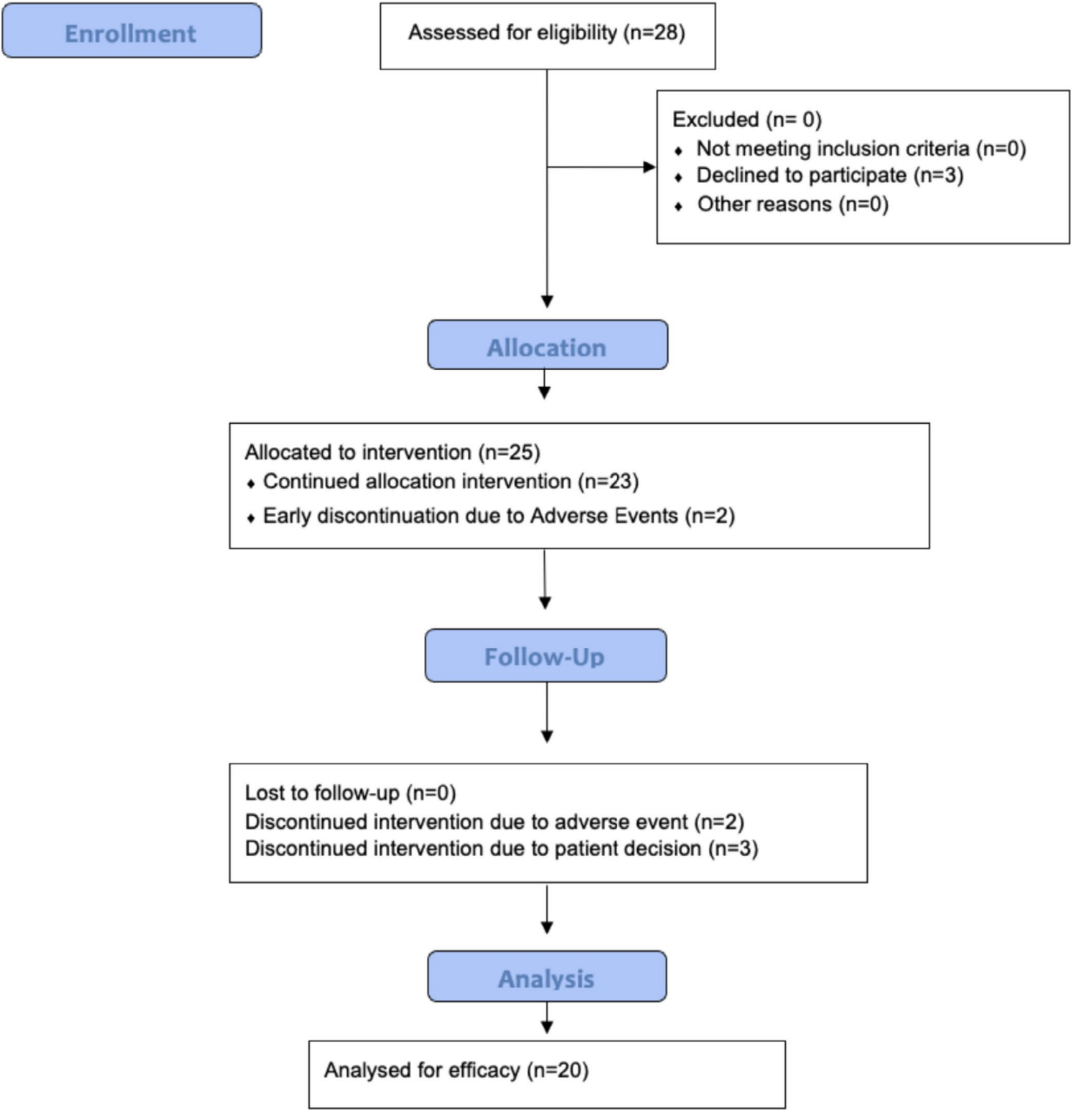
CONSORT flow diagram of the KGF‐HS trial.

Between April 27, 2020, and June 24, 2022, 28 patients were enrolled and consented for the KGF‐HS clinical trial (Figure 1). All patients were recruited at the University of Arizona Cancer Center medical oncology clinic and all protocol visits were coordinated with the patient's scheduled chemotherapy appointments. This was an open‐label, non‐blinded study as all patients were enrolled in the single‐arm prospective cohort. The baseline demographic data of the patients are summarized in Table 1. 85% of evaluable patients in the study received taxane‐based chemotherapy, whereas 15% were given anthracycline‐based chemotherapy. In the baseline group, the mean age was 60 years.

**TABLE 1:** Demographic information.

Parameter	Patients consented (*n* = 28)	Patients evaluable (*n* = 20)
Age, years		
Median (range)	63 (38–78)	60 (38–77)
Race, *N* (%)		
White	24 (86)	16 (80)
Other	4 (14)	4 (20)
Ethnicity		
Hispanic or Latino	2 (7)	1 (5)
Non‐Hispanic	26 (93)	19 (95)
Planned chemotherapy regimen, *N* (%)		
Anthracycline‐based regimen	6 (21)	3 (15)
Taxane‐based regimen	22 (79)	17 (85)

KGF‐HS application occurred twice daily starting 48 h before the first dose of chemotherapy and continued throughout the entire chemotherapy regimen. At baseline and before each chemotherapy cycle, patients had an alopecia assessment by two trained study team members who performed that assessment independently. The alopecia assessment was made using the Common Terminology Criteria for Adverse Events 4.0 (CTCAE v4.0) grading system. Patients also performed their own alopecia assessment and were asked about wig and scarf use after each cycle using the CTCAE v4.0 grading system and the alopecia pictorial tool. The European Organization for Research and Treatment of Cancer Quality of Life Questionnaire‐Core 30 (EORTC QLQ‐C30), Hospital Anxiety and Depression Scale (HADS), Body Image Scale (BIS), and comfort scale questionnaires were completed by participants at baseline, after every cycle of chemotherapy until the completion of four cycles, and at completion of chemotherapy if the participant received more than four cycles of chemotherapy. After completion of treatment, patients were revisited at 1, 3, and 6 months to measure hair regrowth and to complete study questionnaires. Patients who had hair loss were given the option to get KGF‐HS as an open label once they had completed their chemotherapy. Patients who had hair preservation were given the option to continue study drug for up to 3 months post chemotherapy.

Quality of life was assessed by the three standard scales: EORTC QLQ‐C30, HADS, and BIS. The emotional functional scale score is calculated from EORTC QLQ Q30 items 21–24 and the social functioning scale is calculated from EORTC QLQ‐C30 items 26 and 27 using the EORTC QLQ‐C30 scoring formula, which linearly transformed the average raw scored to a range from 0 to 100 (a higher score represented a higher level of functioning). A change in the functioning scale score of 5–10 was considered “a little” change for better or worse, a change of greater than 10–20 was considered a “moderate” change, and a change greater than 20 was considered a “very much” change. The HADS was used to assess anxiety and depression. It includes seven questions to assess anxiety and seven to assess depression. The summary scores (sum of the seven question items) for anxiety and depression range from 0 to 21: scores of 0–7 are considered normal, 8–10 are considered borderline abnormal (borderline case), and 11–21 are considered abnormal (case). The BIS summary score is the sum of the first 9 (of 10) items in the BIS. The last item in the BIS for scar is not applicable to the study participants, so it is not included. The summary score ranges from 0 to 27; a score of 0 indicates no symptoms or distress, and a higher score indicates increasing symptoms or distress. For all three questionnaires, missing items were replaced by the average of non‐missing items.

The main study end point is hair preservation following chemotherapy. Preservation success was defined as CTCAE v4.0 alopecia grade 0 (no loss) or Grade 1 (< 50% loss, not requiring a wig). Failure was defined as Grade 2 (≥ 50% loss, requiring a wig). Patients completing at least one chemotherapy cycle were included in the overall response analysis. Independent, blinded clinicians assessed the primary efficacy end point. Participant withdrawals were considered treatment failures. Secondary end points included EORTC QLQ‐30, HADS, BIS, and comfort questionnaire scores.

The primary safety end point was adverse events connected to the KGF‐HS usage. An adverse event is any undesirable sign, symptom, medical condition, or experience that develops or worsens in severity due to the serum treatment. Patient adverse events will be reported from the start of the study hair serum throughout the study and 30 days after the last use of study serum. Patients who experience an ongoing adverse event related to the treatment beyond 30 days will continue to be contacted by a member of the study team as needed. Secondary safety end points included a participant‐reported comfort scale categorized in five levels: very uncomfortable, uncomfortable, comfortable, reasonably comfortable, and very comfortable.

### 2.1 Statistical Methods

The statistical methods were based on a power calculation that determined a minimum of 20 patients were needed for the trial. The decision rule is based on the lower bound of a one‐sided 95% confidence interval; at least 4 out of 20 patients with response results in a lower bound greater than 5% success. The proportion with success and the lower bound of a one‐sided exact confidence interval will be computed. If more than four positive responses are noted, then part 2 of the trial would be initiated. Part 2 of the study included a randomized double‐blind placebo‐controlled study. Comparison of differences in means of secondary end points including quality of life scores was performed using two sample independent *t*‐tests.

## 3 Results

Of the 28 patients, 20 completed chemotherapy and were evaluable for the primary end point. Of the eight patients that did not complete the trial, five withdrew consent prior to starting the KGF‐HS—three due to patient decision and two due to adverse events. During the trial, two patients had adverse events and withdrew. The adverse events due to KGF‐HS were Grade 1 pruritus (two patients). Once patients reached Grade 2 alopecia, the KGF‐HS treatment was stopped.

Of the patients who completed the study, 0 of 20 women preserved their hair (Table 2). The mean duration of the KGF‐HS treatment was 4.6 weeks (range 2–10 weeks). Due to less than four positive responses noted, the trial was closed based on statistical design and Part 2 of the trial was not initiated.

**TABLE 2:** Summary of hair preservation.

Parameter	Chemotherapy (*n* = 20)
Hair preservation	
Success	0
Alopecia grade 0	
Alopecia grade 1	
Failure	20

Secondary end points included quality of life assessments that were taken at baseline and after every cycle of chemotherapy. The change in emotional and social functioning scores for patients (*n* = 20) undergoing treatment was not significant (Table 3). The baseline and chemotherapy HADS anxiety and depression summary scores were normal (< 7) at baseline and after chemotherapy. The median BIS summary score was 2.5 at baseline and 5 after chemotherapy.

**TABLE 3:** Quality‐of‐life questionnaire scores.

Parameter	Baseline (*n* = 20)	End of treatment (*n* = 20)
EORTC QLQ‐C30 scores, median (range)		
Emotional functioning	6 (4–14)	6 (4–12)
Social functioning	2 (2–5)	3 (2–6)
HADS score, median (range)		
Anxiety	5.5 (0–15)	5 (0–13)
Depression	1 (0–4)	2 (0–9)
BIS score, median (range)	2.5 (0–21)	5 (1–24)

Abbreviations: BIS, Body Image Scale questionnaires; EORTC QLQ‐C30, The European Organization for Research and Treatment of Cancer Quality of Life Questionnaire‐Core 30; HADS, Hospital Anxiety and Depression Scale.

For the patients who continued the trial post chemotherapy (*n* = 3), there was no significant difference in the emotional and social functioning score. The baseline HADS anxiety score for the post chemotherapy patients was borderline abnormal at an 8 while the depression score was normal (< 7). The anxiety score decreased to normal after 6 months, whereas the depression score had no significant difference. The median BIS summary score underwent a decrease from baseline to 6 months post chemotherapy. The median score at baseline was 13 while after 6 months it decreased to 1.

Adverse events related to hair serum were collected, whereas reactions to chemotherapy were not collected. There were two related adverse events, with both being Grade 1 pruritus. All adverse events resolved after discontinuing hair serum. The majority of patients were comfortable, reasonably comfortable, or very comfortable during the hair serum treatment according to the comfort scale. The one patient who reported uncomfortable withdrew from the trial due to Grade 1 pruritus.

## 4 Discussion

Current treatment options for CIA are limited. Scalp cooling has been shown to reduce CIA, but in practice there are time and financial barriers that limit its use [4]. KGF is a member of the fibroblast growth factor family that specifically binds to the KGF receptor, a tyrosine kinase [6]. High levels of KGF and the KGF receptor have been found on the skin, with the mRNA localized exclusively to the dermis [6]. KGF has been identified as activating multiple downstream pathways, including MAPK and PI3K, that promote proliferation, development, and differentiation of many epithelial cells, including keratinocytes in epidermal, hair follicle, and sebaceous gland cells [6]. KGF has been shown to be upregulated in wound healing, psoriasis, and epithelial regeneration. It has also been shown to increase hair follicle resistance to toxic insult in preclinical models, have a cytoprotective effect on hair cells, and increase regeneration of epithelial cells post toxic exposure [6, 7]. Currently, KGF is used to prevent oral mucositis in chemotherapy patients, as it increases epithelial cell resistance to toxic insult [8]. In mouse models, KGF has been shown to preserve hair follicles in as much as 50% when beginning 1 day prior to the onset of chemotherapy [6].

Another topical agent which has been evaluated for CIA is calcitriol, the hormonally active form of vitamin D. Calcitriol activates a nuclear transcription factor VDR that modifies gene transcription and differentiation of keratinocytes, decreases inflammatory cytokines, and promotes the anagen phase. Overall, vitamin D produces anti‐inflammatory and immunomodulatory properties that can regulate keratinocyte differentiation and proliferation [9]. Vitamin D deficiency has been strongly associated with various forms of alopecia [9, 10]. In a study of topical calcitriol in 23 patients with breast or gynecologic cancers undergoing taxane‐based chemotherapy, hair loss less than 50% from baseline was reported in eight participants at Week 7, of which two participants maintained more than 50% of their hair by Week 15 [11]. Other potential agents such as topical epinephrine are being studied. Epinephrine is a vasoconstrictor that was demonstrated to be effective in preventing CIA and oral mucositis in animal models [12]. Specifically, epinephrine binds to adrenergic G‐protein‐coupled receptors, activating the adenylate cyclase pathway causing vasoconstriction in scalp microvasculature [12]. Topical application of epinephrine to the dorsal skin of neonatal rats resulted in 95% coat retention in those treated with *N*‐nitroso‐*N*‐methylurea, and 16% coat retention in rats receiving systemic cyclophosphamide indicating a significant protective effect against alopecia [12]. The distinct biological mechanism of KGF as a fibroblast growth factor protein and its unique properties of hair preservation after toxic insult made it a novel and promising approach for CIA prevention.

This open‐label, single‐arm clinical trial evaluated the potential efficacy of topical KGF‐HS for the prevention of CIA. All 20 patients who were evaluable for the primary end point had Grade 2 alopecia (> 50% hair loss) and discontinued the KGF‐HS. Interestingly, the quality‐of‐life measures that asked about emotional, social, and body image domains had no significant change despite complete hair loss. The majority of patients tolerated the KGF‐HS well and reported the application to be comfortable. The two related adverse events of pruritus resolved after stopping KGF‐HS. Although a reported incidence has not been published, pruritus and rash are the most likely expected AEs of KGF‐HS (as it is a topical application). The major limitation of our study is that it is an open‐label, single‐arm study with a small sample size. In addition, we did not evaluate different administration schedules or dosages, which limited our ability to accurately assess whether KGF‐HS is truly ineffective in preventing CIA.

The existing evidence on KGF includes mouse models demonstrating hair follicle preservation after cytotoxic events and increased hair count in human participants after intradermal injections [13]. There are no published studies directly assessing KGF in participants undergoing chemotherapy, making this trial important in assessing its potential. Our results failed to demonstrate that KGF is biologically effective in preventing CIA. While KGF‐HS is well tolerated, it is not an efficacious intervention for preventing CIA in its current formulation and delivery method. Potential reasons could be the bioavailability of the current formulation, duration it was applied prior to cytotoxic treatment, and/or potency of the cytotoxic agents used in our study.

CIA continues to be clinically important for women with breast cancer. Barriers for widespread use and implementation of scalp cooling devices are due to patient constraints (financial and additional time added on for infusion) and treatment facility challenges (increased infusion time leads to a smaller number of patients being treated). Patients' ease of use and acceptability of a topical agent to prevent CIA supports the development of additional solutions that are affordable and timely. Future research should aim to optimize the delivery and bioavailability of KGF to adequately assess its therapeutic potential. Advances in transdermal and targeted delivery technologies such as laser‐assisted drug delivery or nanocarrier systems (liposomes or hydrogels) could provide controlled and sustained release, extending the duration of bioactivity [13]. In conclusion, our study indicates that topically applied KGF is not an effective treatment option for the prevention of CIA in its current formulation. However, for a definitive conclusion about the effectiveness of KGF in this indication, new studies with different formulations and combinations and using advanced transdermal and targeted delivery technologies such as laser‐assisted drug delivery or nanocarrier systems (liposomes or hydrogels) are needed.

## Author Contributions

Katherine Mann and Preethika Potluri were involved in data analysis, manuscript drafts, and reviewed and approved the final manuscript. Emma E. Paul prepared manuscript drafts, and reviewed and approved the final manuscript. Jennifer M. Segar and Sima Ehsani contributed to patient enrollment, manuscript drafts, and review and approval of the final manuscript. Denise Roe contributed to study design, data analysis, and review and approval of the final manuscript. Pavani Chalasani contributed to study design, patient enrollment, data analysis, manuscript drafts, and review and approval of the final manuscript.

## Ethics Statement

The study was approved by the University of Arizona institutional review board in April 2020 and registered on clinicaltrials.gov (NCT04554732). The trial was also conducted in accordance with the Declaration of Helsinki for biomedical research involving human subjects and local regulatory requirements. All included patients gave written informed consent for clinical trial participation in compliance with the informed consent regulations in 21 CFR 50.

## Conflicts of Interest

The authors declare no conflicts of interest.

## Data Availability

The data that support the findings of this study are available on request from the corresponding author. The data are not publicly available due to privacy or ethical restrictions.

## References

[1] R. M. Trueb , “Chemotherapy‐Induced Hair Loss,” Skin Therapy Letter 15 (2010): 5–7. 20700552

[2] M. Ross and E. Fischer‐Cartlidge , “Scalp Cooling: A Literature Review of Efficacy, Safety, and Tolerability for Chemotherapy‐Induced Alopecia,” Clinical Journal of Oncology Nursing 21, no. 2 (2017): 226–233, 10.1188/17.CJON.226-233. 28315539

[3] B. Benjamin , D. Ziginskas , J. Harman , and T. Meakin , “Pulsed Electrostatic Field (ETG) to Reduce Hair Loss in Women Undergoing Chemotherapy for Breast Cancer: A Pilot Study,” Psycho‐Oncology 11 (2002): 244–248. 12112485 10.1002/pon.593

[4] J. Nangia , T. Wang , C. Osborne , et al., “Effect of a Scalp Cooling Device on Alopecia in Women Undergoing Chemotherapy for Breast Cancer: The SCALP Randomized Clinical Trial,” Journal of the American Medical Association 317, no. 6 (2017): 596–605, 10.1001/jama.2016.20939. 28196254

[5] J. Uscher , “Cold Caps and Scalp Cooling Systems,” Breastcancer.org, https://www.breastcancer.org/treatment‐side‐effects/hair‐loss/cold‐caps‐scalp‐cooling#:~:text=Covering%20the%20cost%20of%20scalp%20cooling,‐Advertisement&text=Cold%20caps%20typically%20cost%20about,a%20full%20course%20of%20chemotherapy.

[6] D. M. Danilenko , B. D. Ring , D. Yanagihara , et al., “Keratinocyte Growth Factor Is an Important Endogenous Mediator of Hair Follicle Growth, Development, and Differentiation: Normalization of the Nu/Nu Follicular Differentiation Defect and Amelioration of Chemotherapy‐Induced Alopecia,” American Journal of Pathology 147, no. 1 (1995): 145–154. 7604876 PMC1869891

[7] C. Barazzone , Y. R. Donati , A. F. Rochat , et al., “Keratinocyte Growth Factor Protects Alveolar Epithelium and Endothelium From Oxygen‐Induced Injury in Mice,” American Journal of Pathology 154 (1999): 1479–1487. 10329601 10.1016/S0002-9440(10)65402-8 PMC1866589

[8] R. Spielberger , P. Stiff , W. Bensinger , et al., “Palifermin for Oral Mucositis After Intensive Therapy for Hematologic Cancers,” New England Journal of Medicine 351 (2004): 2590–2598. 15602019 10.1056/NEJMoa040125

[9] K. Saini and V. Mysore , “Role of Vitamin D in Hair Loss: A Short Review,” Journal of Cosmetic Dermatology 20, no. 11 (2021): 3407–3414, 10.1111/jocd.14421. 34553483

[10] A. F. Rashad , E. Elgamal , and I. Fouda , “Intralesional Vitamin D3 in Treatment of Alopecia Areata: A Randomized Controlled Clinical Trial,” Journal of Cosmetic Dermatology 21, no. 10 (2022): 4617–4622, 10.1111/jocd.14844. 35152536

[11] M. E. Lacouture , H. Dion , S. Ravipaty , et al., “A Phase I Safety Study of Topical Calcitriol (BPM31543) for the Prevention of Chemotherapy‐Induced Alopecia,” Breast Cancer Research and Treatment 186, no. 1 (2021): 107–114, 10.1007/s10549-020-06005-6. 33206291 PMC8388155

[12] C. M. Soref and W. E. Fahl , “A New Strategy to Prevent Chemotherapy‐ and Radiotherapy‐Induced Alopecia Using Topically Applied Vasoconstrictor,” International Journal of Cancer 136, no. 1 (2015): 195–203, 10.1002/ijc.28961. 24811525 PMC4342350

[13] T. C. Wikramanayake , N. I. Haberland , A. Akhundlu , A. Laboy Nieves , and M. Miteva , “Prevention and Treatment of Chemotherapy‐Induced Alopecia: What Is Available and What Is Coming?,” Current Oncology 30, no. 4 (2023): 3609–3626, 10.3390/curroncol30040275. 37185388 PMC10137043

